# Giving drought the cold shoulder: a relationship between drought tolerance and fall dormancy in an agriculturally important crop

**DOI:** 10.1093/aobpla/plu012

**Published:** 2014-03-24

**Authors:** Keith G. Pembleton, Puthigae Sathish

**Affiliations:** 1 Tasmanian Institute of Agriculture, University of Tasmania, PO Box 3523, Burnie, TAS 7320, Australia; 2 Pastoral Genomics, c/o ViaLactia (NZ) Ltd, PO Box 109185, Newmarket 1149, New Zealand

**Keywords:** Alfalfa, forage legumes, gene expression, lucerne, moisture stress.

## Abstract

The growth of fall dormant/freezing tolerant plants often surpasses the growth of non-fall dormant/non-freezing tolerant types of the same species under water-limited conditions, while under irrigated conditions non-fall dormant types exhibit superior yield performance. To investigate the mechanism behind this phenomenon, we exposed seven diverse alfalfa (*Medicago sativa*) cultivars to water-limited and fully watered conditions and measured their shoot growth, shoot water potential and gas exchange parameters and the relative abundance of taproot RNA transcripts associated with chilling stress/freezing tolerance. Fall dormant cultivars had greater shoot growth relative to the fully watered controls under a mild water deficit (a cumulative water deficit of 625 mL pot^−1^) and did not close their stomata until lower shoot water potentials compared with the more non-fall dormant cultivars. Several gene transcripts previously associated with freezing tolerance increased in abundance when plants were exposed to a mild water deficit. Two transcripts, *corF* (encodes galactinol synthase) and *cas18* (encodes a dehydrin-like protein), increased in abundance in fall dormant cultivars only. Once water deficit stress became severe (a cumulative water deficit of 2530 mL pot^−1^), the difference between fall dormancy groups disappeared with the exception of the expression of a type 1 sucrose synthase gene, which decreased in fall dormant cultivars. The specific adaptation of fall dormant cultivars to mild water deficit conditions and the increase in abundance of specific genes typically associated with freezing tolerance in these cultivars is further evidence of a link between freezing tolerance/fall dormancy and adaption to drought conditions in this species.

## Abstract (precis)

Fall dormant/freezing tolerant plants often also exhibit superior tolerance to drought conditions compared to their non-fall dormant/freezing intolerant counterparts. This experiment aimed to investigate this phenomenon in an agriculturally important crop. Seven alfalfa cultivars with varying levels of fall dormancy/freezing tolerance were exposed to a water deficit. The more fall dormant cultivars had superior tolerance to a mild water deficit. Two genes, CAS18 (encodes for a dehydrin like protein) and CorF (encodes for a galactinol synthase), were up regulated in association with this drought tolerance. Both these genes are early response genes, providing clues to the stress signalling pathways involved.

## Introduction

Drought is a major limitation to agricultural production. Drought causes a cessation of plant growth and in extreme cases leads to the senescence of part or the whole of plants. There is a diverse range of adaptations to water-limited conditions within the plant kingdom, and environments that may be extreme for one species may be relatively benign for another. Adaptation to and tolerance of water deficit stress is often associated with adaptation to and tolerance of freezing temperatures as both environmental conditions lead to osmotic and oxidative stresses at a cellular level (Thomashow 1999; Zhu 2002) and disrupt plant water status (Chinnusamy *et al.* 2004). Cross-talk between the stress signalling pathways involved in both environmental stresses is a common phenomenon (Chinnusamy *et al.* 2004) and often the same or similar metabolites accumulate in response to both stresses (Kaplan *et al.* 2004; Rizhsky *et al.* 2004; Usadel *et al.* 2008; Urano *et al.* 2009).

Alfalfa (syn. lucerne; *Medicago sativa*) is a globally important forage legume crop with a total area of production of ∼32.5–35 million hectares worldwide (Russelle 2001; Radovic *et al.* 2009). Its importance is further increased by its ability to fix atmospheric nitrogen (through a symbiotic relationship with the rhizobia *Sinorhizobium* (*Ensifer*) *meliloti*), making it an important source of nitrogen within agricultural production systems (Vance *et al.* 1988). Based on their level of fall/winter growth, alfalfa cultivars can be separated into four fall dormancy groups: fall dormant, semi-fall dormant, non-fall dormant and highly non-fall dormant (Barnes *et al.* 1988). This in turn can be related back to the genetic pedigree of a cultivar (Barnes *et al.* 1988; He *et al.* 2009), with non-fall dormant types entirely comprised of ssp. *sativa* and fall dormant types containing some spp. *falcata* in their background. As part of the characterization process of new alfalfa cultivars, they are scored on a scale of 1–11, with 1 assigned to the most fall dormant cultivars and 11 assigned to the most non-fall dormant cultivars, based on their shoot length 1 month after defoliation in late autumn (Teuber *et al.* 1998). This score strongly correlates to the level of plant injury when the cultivar is exposed to freezing temperatures (Sheaffer *et al.* 1992; Schwab *et al.* 1996) and consequently is an important consideration when selecting cultivars to be grown in environments that expose plants to sub-zero temperatures over the winter.

A consistent trend in alfalfa field experiments undertaken in the cool temperate regions of Australia under water-limited conditions is the superior yield performance of fall dormant cultivars of alfalfa compared with non-fall dormant cultivars (Pembleton *et al.* 2010*a*, *b*). This is despite there being no difference in the soil water use pattern between cultivars under such conditions (Pembleton *et al.* 2011). Interestingly, in these environments there is no need for adaptation to freezing temperatures over the winter and when water deficit is alleviated non-fall dormant cultivars outperform or at least equal the yield of fall dormant cultivars (Pembleton *et al.* 2010*a*, *b*). Adaptation of fall dormant cultivars of alfalfa to water-limited conditions has been noted in the literature for some time (e.g. Grandfield 1943). When exposed to a water deficit, fall dormant and semi-fall dormant cultivars have been identified as being able to maintain a higher shoot water potential (Grimes *et al.* 1992; Pembleton *et al.* 2009), greater rates of stomatal conductance (Hattendorf *et al.* 1990) and taproot carbohydrate reserves (Boschma and Williams 2008) compared with non-fall dormant and highly non-fall dormant cultivars.

Many reports have highlighted the similarities between gene families (including dehydrins, protein kinases and transcription factors) that are upregulated in plants exposed to cold temperatures and water deficits (see Chinnusamy *et al.* 2004; Beck *et al.* 2007; Krasensky and Jonak 2012 for reviews). Transgenic modification of alfalfa with a superoxide dismutase from *Nicotiana plumbaginifolia* Viv. (McKersie *et al.* 1993, 1996, 1999) or a trehalose from yeast (Suarez *et al.* 2009) has improved tolerance to both freezing and water deficit stress. Furthermore, the alfalfa gene transcripts *cas17* (Wolfraim and Dhindsa 1993), *cas18* (Wolfraim *et al.* 1993) and CAR1/*cor*15 (Laberge *et al.* 1993) have been noted to increase in abundance when plants are exposed to either water deficit or freezing stress. In the field, it has been identified that the expression of freezing tolerance/fall dormancy, nitrogen storage and carbon partitioning genes during late autumn is enhanced by exposure to water deficit conditions over summer (Pembleton *et al.* 2010*c*). Clearly, several candidate genes may contribute to the superior performance of fall dormant cultivars when exposed to water deficits. To date, no attempt has been made to screen these genes to determine which are responsible for this specific adaptation. Given that alfalfa cultivars can be easily classified into a quantitative ranking of freezing tolerance based on their fall dormancy rating and that fall dormant cultivars exhibit superior adaptation to water-limited conditions compared with non-fall dormant and highly non-fall dormant cultivars, it is an ideal species to assess and explore the linkage between freezing tolerance and drought tolerance in perennial forages. This paper reports on a study undertaken to investigate the adaptation of seven alfalfa cultivars/lines representing a fall dormancy score from 3 (dormant) to 10 (highly non-fall dormant) to a water deficit and the expression profile of select genes. The aim of these experiments was to determine what level of fall dormancy is required to achieve superior tolerance to water deficit stress and to establish the relationship between the expression of genes known to be cold inducible or associated with freezing tolerance and the response of cultivars with differing levels of fall dormancy to a water deficit.

## Methods

### Plant material

Bare (uncoated/untreated) seeds of alfalfa cultivars were sourced from commercial seed companies. These cultivars represented a broad range of fall dormancy (FD) ratings and were Q31 (FD rating of 3; Seed Distributors, Wingfield, SA, Australia), Grasslands Kaituna (FD rating of 4.5; Wrightson Seeds Australia, Truganina, VIC, Australia), A5225 (FD rating of 5; Cal/West Seeds, Woodland, CA, USA), SARDI 7 (FD rating of 7; Heritage Seeds, Mulgrave, VIC, Australia), Sequel HR (FD rating of 9; Wrightson Seeds Australia) and SARDI 10 (FD rating of 10; Heritage Seeds). In addition, an experimental line FFI DT1 (putative FD rating of 2; SARDI, Urrbrae, SA, Australia) was also included.

### Screening test of fall dormancy

**Figure 1.**
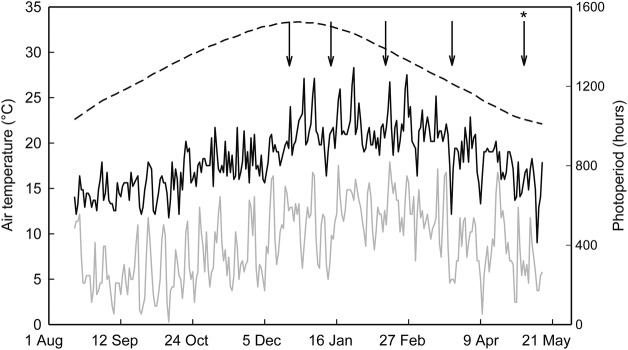
Daily maximum (black line) and minimum (grey line) air temperatures and photoperiod (broken line) at the Cradle Coast Campus of the University of Tasmania in Burnie, TAS, Australia from August 2011 to May 2012. Arrows indicate when the pots of alfalfa used in the fall dormancy screening test were defoliated. The arrow marked with the asterisk indicates when shoot length was assessed.

As plant material was sourced from a range of herbage development programmes, there was a need to standardize their fall dormancy classifications. Consequently, plants were grown outdoors, during the southern hemisphere autumn/fall and winter months, in 6-L (337 mm high, 75 mm radius) pots containing a proprietary potting mix (Earthcore all-purpose potting mix; Van Schaik's Bio-Gro Pty Ltd, Mt Gambia, SA, Australia) at the Cradle Coast Campus of the University of Tasmania in Burnie, TAS, Australia (41.06°S, 145.88°E, 100 m above sea level). Pots were placed on wooden frames elevated 150 mm above the ground. Five seeds of each cultivar were sown into four replicates of pots on 29 August 2011. Pots were arranged in a randomized complete block design. At the emergence of the first unifoliate leaf at 14 days after sowing (DAS), plants were spray inoculated with rhizobia bacteria (*S.* (*E.*) *meliloti*, Group AL; Becker Underwood Pty Ltd, Somersby, NSW, Australia) followed by a hand watering to wash the inoculant into the soil. At the emergence of the third trifoliate leaf (30 DAS), each pot was thinned by hand to one plant per pot. Plants were then grown until 24 March 2012 and were defoliated to a height of 50 mm each time crown bud elongation was observed to occur, giving a total of four defoliations (Fig. 1). Immediately after defoliation each pot received 2.7 g of slow-release fertilizer (Osmocote^®^; Scotts Australia Pty Ltd, Baulkham Hills, NSW, Australia; 19.4 % N, 1.6 % P, 5 % K, 9 % S, 1.8 % Fe, 0.5 % Ca, 0.5 % Mg, 0.3 % Mn, 0.1 % Cu, 0.1 % Zn, 142 mg kg^−1^ B and 90 mg kg^−1^ Mo). Similar to the standard test to characterize fall dormancy in alfalfa (Teuber *et al.* 1998), plants were defoliated on 24 March 2012 and were grown for another 42 days (5 May 2012). On this day the length of two shoots per plant was measured to assess the level of fall dormancy. Daily maximum and minimum temperatures and photoperiod (Fig. 1) were monitored and recorded using a weather station (HOBO weather station; Onset Computer Corp., Bourne, MA, USA). Pots were watered twice daily with a drip irrigation system to ensure that water supply did not limit plant growth.

### Water deficit experiment

#### Growing conditions

The water deficit experiment was undertaken within the glasshouse research facility of the Tasmanian Institute of Agriculture at the Cradle Coast Campus of the University of Tasmania in Burnie, TAS, Australia. Within the glasshouse, air temperature was maintained at an average of 22 °C during the day (ranging between 25 and 18 °C) and at an average of 10 °C during the night (ranging between 14 and 8 °C). Photoperiod was extended beyond the natural day-length to 14 h with the aid of three 400-W halogen lamps suspended 2 m above the plant canopy, covering an area of 7 m × 7 m. These growing conditions were maintained with the aid of a computer-based glasshouse control system (Priva Maximizer; Priva Computers Inc., Vineland Station, ON, Canada). Temperatures and day-length were monitored using sensors and a data logger (HOBO microstation data logger and sensors; Onset Computer Corp.) separate to the glasshouse environmental control system.

Approximately 16 viable seeds were sown into 10-L (340 mm high, 96 mm radius) polyethylene bag pots containing 13.2 kg of dry sandy soil sourced from a local landscaping supplier. This soil had a baseline chemical fertility of 14 mg kg^−1^ of P (Olsen extraction; Olsen *et al.* 1954), 140 mg kg^−1^ of K (Colwell extraction; Colwell 1963), 0.15 mg kg^−1^ of Cu, 100 mg kg^−1^ of Fe, 5.6 mg kg^−1^ of Mn, 3.1 mg kg^−1^ of Zn (DTPA extraction; Lindsey and Norvell 1978) and 6.8 mg kg^−1^ of S (KCl40 extraction; Barrow 1967). The soil had a volumetric soil water content of 0.11 mm mm^−1^ at a soil water potential of −10 kPa. To address soil fertility limitations, each kilogram of soil received 0.19 g of Osmocote^®^ (Scotts Australia Pty Ltd), 0.13 g of triple super phosphate (21 % P, 1 % S) and 0.08 g of muriate of potash (50 % K). Immediately following sowing, pots were watered by hand until water was observed to drain through the pot. Emergence was observed at 4 DAS. At 8, 14 and 21 DAS, plants were spray inoculated with rhizobia (*S.* (*E.*) *meliloti*, Group AL; Becker Underwood Pty Ltd) followed by a hand watering to wash the inoculant into the soil. At 28 DAS, plants were thinned by hand to eight plants per pot. From then on water was applied through a purpose-built drip emitter irrigation system designed and calibrated to deliver water to each pot at a rate of 2 ± 0.02 L h^−1^. Plants were grown until 65 DAS and then defoliated to 50 mm. Plants were allowed to grow for a further 28 days (93 DAS) before they were once again defoliated to 50 mm. At this point the watering treatments commenced. Plants were grown for a further 35 days to the conclusion of the experiment (128 DAS). Following both defoliations, each pot was fertilized with 2.5 g of Osmocote^®^, 1.7 g of triple super phosphate and 1.0 g of muriate of potash.

#### Experimental design

**Figure 2.**
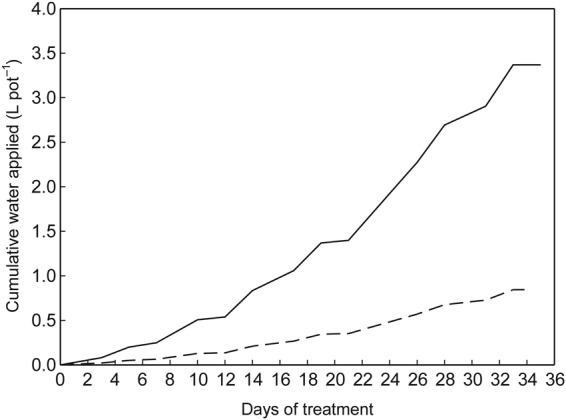
Cumulative water applied to the pots that received 100 % of their water requirement (solid line), and the pots that received 25 % of their water requirement (broken line) over the 35 days of water-deficient treatment.

The experiment was arranged as a completely randomized block design, with four replications. Blocking was based on a glasshouse bench and a buffer of pots of fully watered alfalfa surrounded each block. The buffer pots were maintained at a similar height to their adjacent experimental pots. To address within-block variation, pots were re-randomized within each block three times (65, 93 and 107 DAS) during the experiment. Each cultivar was exposed to two levels of water application, either 100 % of the plants' water requirement or 25 % of the plants' water requirement. Water requirement was determined as the amount of water required to return the pots receiving 100 % of their replacement water requirement to a soil water content of 0.11 mm mm^−1^. Soil water content of the pots was determined at 1- to 2-day intervals by measurement with a theta probe (Delta-T Devices Ltd, Cambridge, UK) connected to a hand-held logger (Infield7; UMS GmbH, Munich, Germany). Soil water content was also determined following each water application. Over the 35 days of water deficit treatment, the pots that received 100 % of their water requirement received 3.37 L of water while the pots that received 25 % of their water requirement received 0.84 L of water (Fig. 2). There were three destructive harvests during the experiment: immediately prior to the experiment commencing, and at 14 and 35 days after the commencement of the water deficit treatments. At each harvest, one of each cultivar by water deficit treatment combination from each replicate was harvested. Excluding the buffer pots, there were a total of 168 pots in the experiment (seven cultivars, two water deficit treatments, three destructive harvests and four replications).

#### Measurements and sample collection

Shoot water potential and gas exchange measurements of plants commenced 3 days prior to the application of water deficit treatments and then occurred at 7-day intervals until the conclusion of the experiment. Measurements occurred between 1000 and 1400 h and were undertaken on plants that were scheduled to be destructively harvested at 35 days after the commencement of watering treatments. Shoot water potential was determined with a pressure chamber (Model 615; PMS Instrument Company, Albany, OR, USA) on the upper 50 mm of two shoots randomly selected per pot. Gas exchange parameters were determined on the middle leaflet on the third most recently emerged leaf on two randomly selected shoots using a portable infrared gas analysis system (LI6400XT; LI-COR Biosciences, Lincoln, NE, USA). Following measurement, the middle leaflet was severed at the petiole and scanned on a flatbed scanner (HP Scanjet 3670; Hewlett-Packard Australia Pty Ltd, Blackburn, VIC, Australia). The resulting image was then analysed using image analysis software (IMAGE J version 1.45h; National Institute of Health, Bethesda, MD, USA) to determine the area of the leaflet. Gas exchange measurements were adjusted based on leaf area, and the net carbon exchange rate and stomatal conductance were then calculated as per the equations outlined in the LI6400XT manual (LI-COR Biosciences).

At each destructive harvest, the pots selected for harvest were defoliated to 50 mm above the soil surface and the number of shoots per pot was counted. The leaves were hand separated from the stems. Total leaf area was determined using a flatbed scanner (HP Scanjet 3670; Hewlett-Packard Australia Pty Ltd) coupled with image analysis software (IMAGE J version 1.45h). Soil was washed free from the roots and crowns. Crowns were cut from the taproot at the lowest crown branch or crown bud. Roots were dried with a paper towel to remove excess water and were then weighed. The uppermost 50 mm of the roots were then diced and snap frozen by immersion in liquid nitrogen before being transferred to a −80 °C freezer. The upper 50 mm of roots were chosen as this was a consistent section of the taproot free of nodules and fibrous roots. The remainder of the root system was then weighed and immediately placed in a −20 °C freezer. Leaves, stems and crowns were dried at 60 °C in a fan-forced oven until a consistent weight was achieved while the roots frozen in the −20 °C freezer were freeze dried for 7 days. Once dry, all material was weighed. The original root dry mass was calculated using the root dry matter content and the original root weight.

#### RNA purification and cDNA synthesis

The taproot material that was snap frozen and stored at −80 °C was ground to a fine powder in liquid nitrogen with a mortar and pestle. Total RNA was extracted from 100 mg of ground material using the Invitrogen TriZol^®^ Plus RNA extraction kit (Life Technologies Australia, Mulgrave, VIC, Australia). The manufacturer's instructions were modified by vortexing of the extraction tube containing the TriZol^®^ reagent, chloroform and sample mixture when the procedure called for shaking by hand and by the addition of a second chloroform phase separation. Following extraction, RNA quantity was determined fluorometrically using a Qubit^®^ 2.0 fluorometer with the broad-range total RNA assay kit (Life Technologies) and RNA integrity was visually checked with ultraviolet light after gel electrophoresis of 1 µg of RNA in 1.5 % agarose gels stained with SYBR^®^ Green (SYBR^®^ safe gel stain; Life Technologies). Following extraction, 20.5 µL of extracted RNA solution was DNase digested using the stringent protocol of the Ambion DNA-free™ kit (Life Technologies Australia). Following the digestion, RNA was re-quantified using the Qubit^®^ 2.0 fluorometer. The absence of genomic DNA contamination was assessed by performing PCRs on 0.35 ng of RNA with forward (5′-GATCAGTGAACTTCGCAAAGTAC-3′) and reverse (5′-AGGGATGCTGCTACTTTGATG-3′) primers designed to amplify a 154 base pair (bp) fragment of the alfalfa acetyl carboxylase gene (Alexander *et al.* 2007; GenBank accession L25042). Products from these PCR reactions were then visually compared with products from PCRs undertaken with alfalfa RNA samples known to contain genomic DNA contamination by electrophoresis in 2.0 % agarose gels stained with SYBR^®^ Green. Any RNA samples that amplified a fragment were re-digested with DNase and re-quantified, and the second PCR products were visualized via gel electrophoresis. Synthesis of cDNA was undertaken using the iScript™ cDNA synthesis kit (Bio-Rad Laboratories Pty Ltd, Gladesville, NSW, Australia) using 0.35 µg of DNase-digested RNA. Following cDNA synthesis, 6 µL from each of the 192 samples was combined to create a ‘calibrator’ sample. This sample was then serially diluted to create 1 : 5, 1 : 50, 1 : 500 and 1 : 5000 dilutions to aid in generating a standard curve required for determining the amplification efficiency of the primer pairs. In addition to this, 2 µL of cDNA synthesized from each sample collected prior to the implementation of water treatments was combined and then diluted 1 : 50.

#### Primer design

Primers were designed for eight genes that were previously identified as having a role in the adaptation of alfalfa to freezing temperatures and eight other genes that were identified as potential reference genes for data normalization (Table 1). Potential reference genes for normalization were chosen from a list of 79 constitutively expressed probe sets identified in Affymetrix chip experiments as part of the *Medicago truncatula* Gaertn gene expression (MtGEA) atlas project (V. A. Benedito, pers. comm.). These probe sets were compared with known alfalfa RNA sequences in GenBank and eight homologues were identified. Primers were designed using the online primer design software ‘Primer 3’ (Rozen and Skaletsky 2000). Criteria for primer design were a PCR product size between 50 and 200 bp, a primer length between 20 and 24 bp, a predicted primer melting temperature between 57 and 59 °C, a GC content between 30 and 80 % and, wherever possible, a large proportion of the target sequence in the 3′ untranslated region of the target transcript. Primers were checked for possible hairpins and self-complementation using the online tool ‘Oligo Calc’ (Kibbe 2007). Primers were custom ordered from a commercial supplier (GeneWorks Pty Ltd, Thebarton, SA, Australia). Primer pairs were tested by performing a PCR on the 1 : 50 dilution of the calibrator sample under the same conditions that would be used for the real-time qRT-PCR analysis of the samples. Following the reaction, PCR products were visualized after electrophoresis in 2 % agarose gels stained with SYBR^®^ Green. Primer pairs that failed to produce a product, produced a product of the incorrect size or produced multiple products were discarded, and new primers were designed and re-ordered.

**Table 1.** Primer pairs (F: forward; R: reverse of the eight reference and eight genes of interest) used in the qPCR analysis of the relative abundance of reference genes (Ref) and genes of interest (GOI) in the taproot tissue of alfalfa cultivars exposed to a water deficit.

Ref or GOI	Gene name	GenBank accession number/reference	Function/annotation	Primer sequence	Predicted fragment size (bp)	Tm (°C)	Region of fragment
Ref	ADP-ribosylation factor	AY466444	Punitive ADP-ribosylation factor	F: TTTCCTGAGTCTGGTGGTTC	109	F: 57.68	UTR: 100 %
				R: ACCCCAAGTAACACTGACGA		R: 58.06	Coding: 0 %
Ref	Phosphoprotein phosphatase type 2A	X70399/Pirck *et al.* (1993)	Phosphoprotein phosphatase	F: TGCTGGATGTCATACTGAGG	98	F: 57.17	UTR: 100 %
				R: CTCCAGAAAGGGTGTCCAG		R: 58.20	Coding: 0 %
Ref	Histone H3	X13674/Wu *et al.* (1989)	Encodes for histone H3 protein	F: CATTCATGCTAAGCGTGTCA	100	F: 58.45	UTR: 25 %
				R: CCCTAACAAAGCGAATCAAC		R: 57.37	Coding: 75 %
Ref	GTP-binding protein	X79278/Jonak *et al.* (1995)	Protein product homologous to small GTP-binding proteins	F: TCCGAAAGTGAGAGAGATACAAA	119	F: 58.14	UTR: 100 %
				R: CCAGCAGCTCCTATTGAAAC		R: 57.58	Coding: 0 %
Ref	Translationally controlled tumour protein	X63872/Pay *et al.* (1992)	Protein product homologous to a translationally controlled human tumour protein	F: ACTACAAGGATGGTGCTGCT	110	F: 57.41	UTR: 36 %
				R: TGATAAATTAGGGGCAGAACA		R: 57.27	Coding: 64 %
Ref	Calmodulin	X52398/Barnett and Long (1990)	Calcium-binding protein	F: ACAGGGCAAATGAGTTTTGA	99	F: 58.20	UTR: 100 %
				R: AACAAACCGACCAACAAAAA		R: 58.03	Coding: 0 %
Ref	Elongation initiation factor	X59441/Pay *et al.* (1991)	Homologue of eukaryotic translation initiation factor 4D	F: TCATTTCTCTAAGCTTTCACATTG	86	F: 57.79	UTR: 100 %
				R: CATAAACACCACCAACACCA		R: 57.71	Coding: 0 %
Ref	Glyceraldehyde-3-phosphate dehydrogenase	GQ398120	Phosphate dehydrogenase	F: TGGAATCGTTGAGGGTCTTA	100	F: 58.15	UTR: 0 %
				R: AGCTCTTCCACCTCTCCAGT		R: 58.06	Coding: 100 %
GOI	Cold acclimation response protein (CAR1/*cas15*)	AF072932/Haagenson *et al.* (2003)	Punitive nuclear regulatory protein	F: GCTTCATCATGTTGAGAGGTG	98	F: 58.30	UTR: 100 %
				R: TTTCTTTCCACACACACACG		R: 58.14	Coding: 0 %
GOI	Bi-modular protein (corC)	L22305/Castonguay *et al.* (1994)	Regulation of plant development	F: GCACGATTGACTTTCACGA	87	F: 58.34	UTR: 100 %
				R: ACAGCGATACACCGTGATTT		R: 58.10	Coding: 0 %
GOI	Cold- and drought-regulated protein (corA)	L03708/Laberge *et al.* (1993)	Cold- and drought-induced protein of unknown function	F: CACCATGCACTCTTTCTCAGT	100	F: 57.94	UTR: 100 %
				R: AACTGAAACTGCTGCACATCT		R: 57.62	Coding: 0 %
GOI	Cold acclimation-specific protein (*cas18*)	L07516/Wolfraim *et al.* (1993)	Cold-induced punitive dehydrin protein	F: TCTGTTTTTGAGTAAGTTGGTTCA	123	F: 58.04	UTR: 100 %
				R: TGCCCCTACACTAAAATTCAA		R: 57.33	Coding: 0 %
GOI	Protein kinase	X82270/Jonak *et al.* (1996)	Stress signalling protein	F: CACTGCTGGGAATTCAATCT	99	F: 57.74	UTR: 100 %
				R: CAACAGAAAGCAGGGTAAGC		R: 57.63	Coding: 0 %
GOI	Galactinol synthase (*corF*)	AY126615/Cunningham *et al.* (2003)	Synthesis of raffinose family oligosaccharides (RFOs)	F: CAGCAATTTTGGAAGCTTATG	95	F: 57.62	UTR: 42 %
				R: AGACGATCATGCGGCTAATA		R: 58.36	Coding: 58 %
GOI	Type 1 sucrose synthase mRNA	AF049487	Synthesis of sucrose	F: ATGCTCTCAAGTACCGCAAA	96	F: 58.53	UTR: 46 %
				R: CGGTTTCTCCATTTCTTCATT		R: 58.17	Coding: 54 %
GOI	High-molecular-weight vegetative storage protein	AF530579/Volenec *et al.* (2002)	Vegetative storage protein	F: ACTTTGATTCCCTCCGTTTT	106	F: 57.64	UTR: 100 %
				R: AGCGCGCAATTCAATTTTA		R: 59.44	Coding: 0 %

#### Determination of gene transcript abundance by real-time qRT-PCR

Real-time qRT-PCR reactions were undertaken in 384-well plates using a Roche 480 LightCycler^®^ PCR system, using SYBR^®^ Green I Master mix (Roche Diagnostics GmbH, Mannheim, Germany). Each reaction contained 5 µL of SYBR^®^ Green I Master mix, 2 µL of a 1 : 50 dilution of template cDNA, 0.5 µL of each primer pair (10 µmol L^−1^ concentrations) and 2 µL of water. Each plate also contained reactions for generating a standard curve for each primer pair, a plate calibrator sample, negative controls and the 1 : 50 dilution of the cDNA collected from each sample that was harvested before the commencement of water deficit treatments. Each reaction was undertaken in triplicate and all liquid handling was undertaken using an automated liquid handling system (epMotion 5075LH; Eppendorf, Hamburg, Germany). The conditions of real-time qRT-PCR reactions were as follows: 95 °C for 5 min, followed by 55 cycles of 95 °C for 10 s, 58 °C for 10 s and 72 °C for 8 s. At the end of the PCR reaction, a melting curve analysis (58–95 °C) was undertaken. LightCycler^®^ 480 software (version 1.5; Roche Diagnostics) was used to determine the crossing point (Cp) values. Cp values and the standard curves were used to determine the concentration of each gene in each sample following adjustment using the plate calibrator sample. Relative concentration values for each of the genes of interest were normalized using the geometric mean of the concentration of the three most stably expressed reference genes identified using the GeNorm version 3.5 software package (Vandesompele *et al.* 2002). Normalized concentration values were divided by the normalized concentration values from the sample generated by taking an equal volume of cDNA from the samples collected prior to the implementation of the water deficit treatments to give a relative concentration.

#### Statistical analysis

Data from the fall dormancy screening test were subjected to an analysis of variance (ANOVA) of a randomized complete block design. Groups of cultivars with similar expression levels of fall dormancy were identified (from here on referred to as fall dormancy groups). Data from the water deficit experiment were subjected to an ANOVA of a randomized complete block design with cultivar, water treatment and harvest date as experimental factors. One-tail *t*-tests were used to identify significant decreases between the water deficit treatment and the fully watered controls of each cultivar and harvest. The relative concentration of each gene of interest from the real-time qRT-PCR analysis was log transformed prior to analysis. To explore the influence of fall dormancy group on the level of water deficit adaption and the abundance of freezing tolerance gene transcripts, growth parameters and gene transcript abundance of the water deficit stressed plants were expressed relative to their corresponding fully watered controls. These relative values were then subjected to an ANOVA with contrasts based on fall dormancy groups. Gene transcript abundance was log transformed prior to analysis. Net carbon exchange rate and stomatal conductance were fitted to the shoot water potential data using a logistic function (Equation 1). The shoot water potential that resulted in a 50 % reduction in carbon exchange rate and a 90 % reduction in stomatal conductance was then estimated from the fitted functions. All statistical analysis was undertaken with the software package GenStat 13th edition (VSN International Ltd, Hemel Hempstead, UK).

(1) $$y = \displaystyle{m \over {1 + \hbox{e}^{b({x - c} )} }}$$

## Results

### Fall dormancy screening test

**Figure 3.**
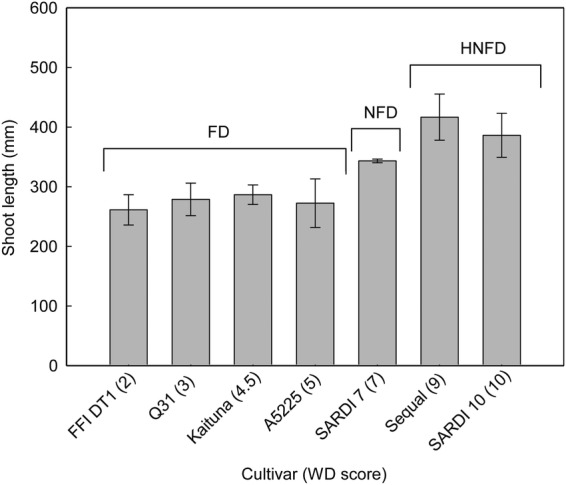
Shoot length (mm) of seven alfalfa cultivars (cultivar fall dormancy score given in parentheses) in late autumn (5 May 2012; 42 days after defoliation). Plants were grown outdoors in pots at Burnie, TAS, Australia. Error bars represent the standard errors of the means (*n* = 4). Cultivars grouped into fall dormant (FD), non-fall dormant (NFD) and highly non-fall dormant (HNFD) groups exhibited no significant difference in shoot length.

Shoot length during autumn/fall when grown outdoors was influenced by cultivar (*P* < 0.05), with a general increase in shoot length with increasing fall dormancy scores (Fig. 3). The cultivars with the longest shoots were Sequel and SARDI 10. The cultivar SARDI 7 also had longer shoots compared with the more fall dormant cultivars of FFI DT1, Q31, Kaituna and A5225. Consequently, FFI DT1, Q31, Kaituna and A5225 were grouped together as the fall dormant group, SARDI 7 as the non-fall dormant group, and Sequel and SARDI 10 as the highly non-fall dormant group.

### Soil water content under water deficit conditions

**Figure 4.**
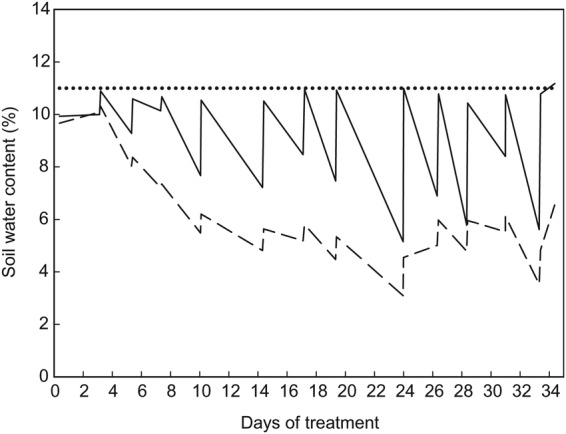
Soil water content in the pots that received 100 % of their water requirement (solid line), and the pots that received 25 % of their water requirement (broken line) over the 35 days of water-deficient treatment. The horizontal dotted line indicates a soil water content of 11 % (equivalent to a soil water potential of −10 kPa for the growing medium).

There was no influence (*P* = 0.39) from cultivar on the soil water content under each of the water treatments. The soil water content of each of the watering treatments differentiated from each other (*P* < 0.001) by 5 days of treatment (Fig. 4). The consistent refilling of the pots to a soil water content of 11 % (equivalent to a soil water potential of −10 kPa for the growing medium) maintained the soil water content in the 100 % treatment throughout the experiment. In contrast, for the 25 % watering treatment after 14 days of treatment the soil water content fluctuated between 6 and 3.5 %.

### DM accumulation under water deficit conditions

Cultivar, water deficit and time of harvest interacted to affect shoot yield (*P* < 0.05). At Day 14 after the beginning of the water deficit treatment, the effect on shoot mass was dependent upon cultivar, with significant differences between plants receiving 100 and 25 % of their water requirement observed in the SARDI 7 and Sequel HR cultivars (Table 2). When cultivars were pooled based on fall dormancy group, the non-fall dormant and highly non-fall dormant groups showed a significant decrease in shoot mass per plant when exposed to a water deficit compared with the fully watered plants. At 35 days of treatment, the plants exposed to a water deficit had a lower shoot yield compared with the fully watered controls for all cultivars, and the same effect was observed when cultivars were grouped based on fall dormancy group. There were interaction effects between water deficit treatment and harvest (*P* < 0.001) and cultivar and water deficit treatment (*P* < 0.01) on total plant mass. Consequently, there was no difference in total plant mass between the plants of each cultivar receiving either 100 or 25 % of their water requirement, at 14 days of water deficit. However, after 35 days of exposure to water deficit treatment, total mass per plant was reduced in all the cultivars compared with their respective controls (*P* < 0.05; Table 2) and the same was noted when cultivars were grouped by fall dormancy groups.

**Table 2.** Shoot mass per plant and total plant mass (g DM plant^−1^) of seven alfalfa cultivars (presented individually and grouped based on fall dormancy grouping) after 14 or 35 days of receiving either 100 or 25 % of their water requirement. One-tail *t*-tests were used to identify decreases between plants receiving 100 or 25 % of their water requirement within a cultivar. Differences are indicated by NS: *P* > 0.05, *P < 0.05, **P < 0.01, ***P < 0.001.

	Shoot mass per plant (g DM plant^−1^)						Total mass per plant (g DM plant^−1^)					
	14 days of drought			35 days of drought			14 days of drought			35 days of drought		
	100 %	25 %	*t*-Test	100 %	25 %	*t*-Test	100 %	25 %	*t*-Test	100 %	25 %	*t*-Test
Cultivar												
FFI DT1	0.27	0.18	NS	0.72	0.24	***	0.99	0.84	NS	2.07	1.21	***
Q31	0.20	0.19	NS	0.85	0.11	***	1.01	0.91	NS	2.28	1.09	***
Kaituna	0.26	0.22	NS	0.74	0.15	***	1.01	0.86	NS	2.28	1.06	***
A5225	0.29	0.19	NS	0.57	0.30	***	0.80	0.84	NS	1.57	1.25	**
SARDI 7	0.31	0.13	*	0.76	0.19	***	1.02	0.81	NS	2.03	1.10	***
Sequel HR	0.33	0.19	*	0.81	0.18	***	1.05	0.81	NS	2.21	1.07	***
SARDI 10	0.30	0.18	NS	0.67	0.19	***	1.01	0.86	NS	1.90	1.06	***
SED	0.0848						0.1576					
Fall dormancy group												
Fall dormant	0.26	0.20	NS	0.72	0.20	***	0.93	0.86	NS	2.05	1.15	***
Non-fall dormant	0.31	0.13	*	0.76	0.19	***	1.02	0.81	NS	2.03	1.10	***
Highly non-fall dormant	0.32	0.19	*	0.74	0.19	***	1.03	0.84	NS	2.06	1.07	***
SED	0.0695						0.1383					

There was no influence from cultivar or fall dormancy group on the shoots per plant, mass per shoot and leaf area per plant nor did cultivar interact with either harvest or water deficit treatment. Harvest and water deficit treatment did interact (*P* < 0.05) to affect these response variables. Shoots per plant, mass per shoot and leaf area per plant did not increase between 14 and 35 days of treatment for the plants that only received 25 % of their water requirement (Table 3). In contrast, shoots per plant, mass per shoot and leaf area per plant increased between Days 14 and 35 of treatment for the fully watered plants.

**Table 3.** Shoots per plant, mass per shoot and leaf area per plant of alfalfa plants (the average of seven cultivars) receiving either 100 or 25 % of their water requirements after 14 and 35 days of treatment.

Days of treatment	Water treatment (%)	Shoots per plant	Mass per shoot (mg shoot^−1^)	Leaf area per plant (m^2^ plant^−1^)
14	100	3.2	88.2	0.046
	25	2.3	80.9	0.024
35	100	3.8	197.5	0.096
	25	2.2	87.0	0.027
SED		0.2	8.8	0.005
*P* value		0.028	<0.001	<0.001

**Figure 5.**
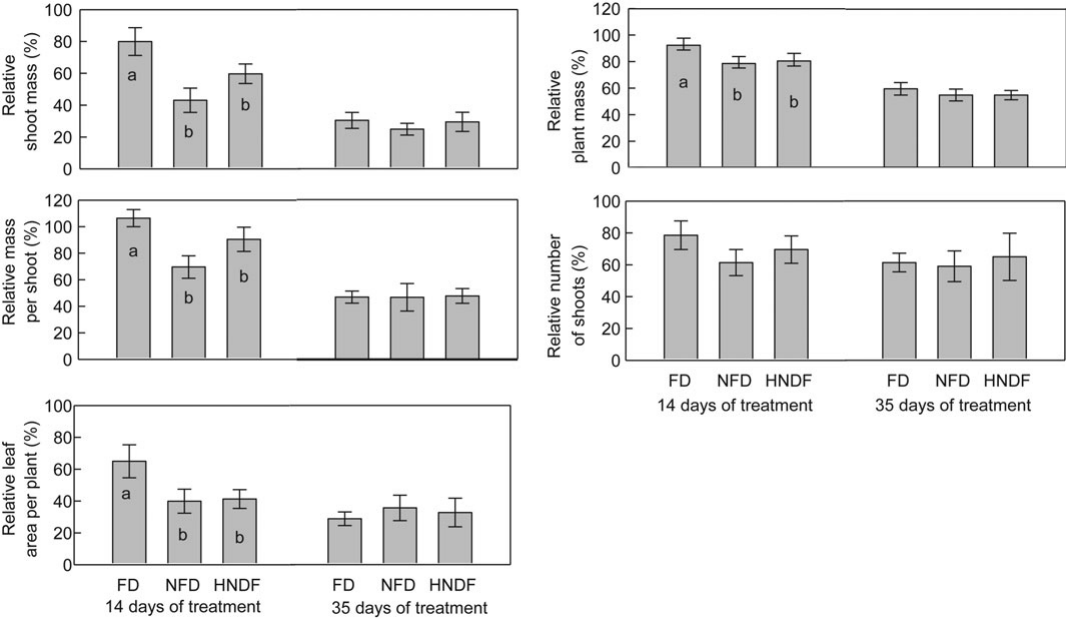
Shoot mass per plant, total plant mass, mass per shoot, number of shoots per plant and leaf area per plant expressed as a proportion of the value achieved by the fully watered controls (relative value) at 14 and 35 days after the initiation of treatments. Cultivars were grouped into fall dormant (FD), non-fall dormant (NFD) and highly non-fall dormant (HNFD) fall dormancy groups based on their level of fall dormancy identified in the fall dormancy screening test. Bars with different letters were identified as being different from each other using ANOVA with non-orthogonal contrasts (*P* < 0.05). Error bars represent the standard errors of the mean (*n* = 4 to 16).

After 14 days of treatment, the cultivars classified into the fall dormant group had a greater (*P* < 0.05) shoot yield, mass per shoot, total plant mass and leaf area per plant relative to the fully watered controls compared with the non-fall dormant or highly non-fall dormant group (Fig. 5). However, this effect was transient and after Day 35 of exposure there was no difference between the fall dormancy groups for these variables.

**Figure 6.**
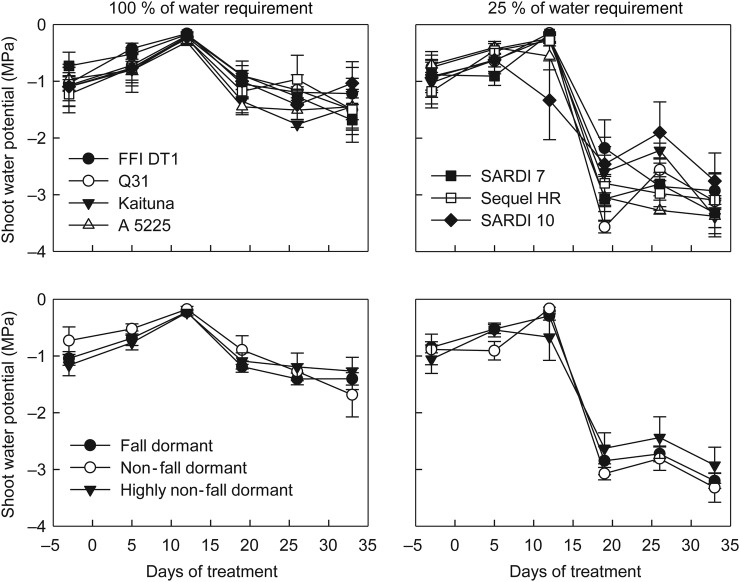
Shoot water potentials of seven alfalfa cultivars (upper panels) and the seven cultivars grouped by their fall dormancy groups (lower panels) through 35 days of receiving either 100 % of their water requirement or 25 % of their water requirement. Error bars represent the standard errors of the mean (*n* = 4).

### Shoot water potential and gas exchange under water deficit conditions

While differences in the shoot water potential occurred between cultivars when exposed to a water deficit, these differences were inconsistent throughout the experiment (Fig. 6). For example, SARDI 10 had the lowest shoot water potential after 12 days of treatment but had the highest shoot water potential after 19 days of treatment. When grouped by fall dormancy, there were no differences (*P* = 0.396) in the shoot water potential within either watering treatments. There was a sharp decline in shoot water potential 19 days after the beginning of the water deficit treatments in all cultivars.

**Figure 7.**
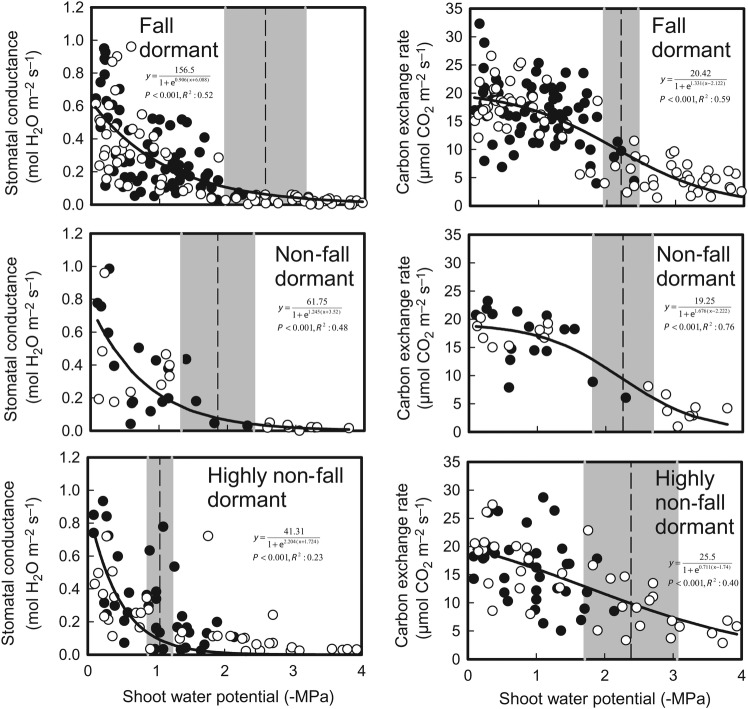
Relationship between shoot water potential and stomatal conductance/carbon exchange rate of alfalfa cultivars grouped into fall dormant, non-fall dormant or highly non-fall dormant dormancy groups. Plants received either 100 % (open circles) or 25 % (closed circles) of their water requirement over 35 days. A logistical function was fitted to the data (solid line). The vertical broken line indicates shoot water potentials at which a 50 % decline in carbon exchange rate and a 90 % decline in stomatal conductance (stomatal closure) occurred. The grey areas represent the standard errors around these estimates.

As shoot water potential decreased, so did net carbon exchange rate and stomatal conductance (Fig. 7). The point of stomatal closure (defined as a 90 % reduction in stomatal conductance) occurred at lower shoot water potentials (−2.54 MPa) in the fall dormant group compared with the highly non-fall dormant group (−1.05 MPa). There was no difference in the shoot water potential that resulted in a 50 % decline in photosynthesis among the fall dormancy groups.

### Real-time qRT-PCR analysis of gene transcript abundance under water deficit conditions

**Figure 8.**
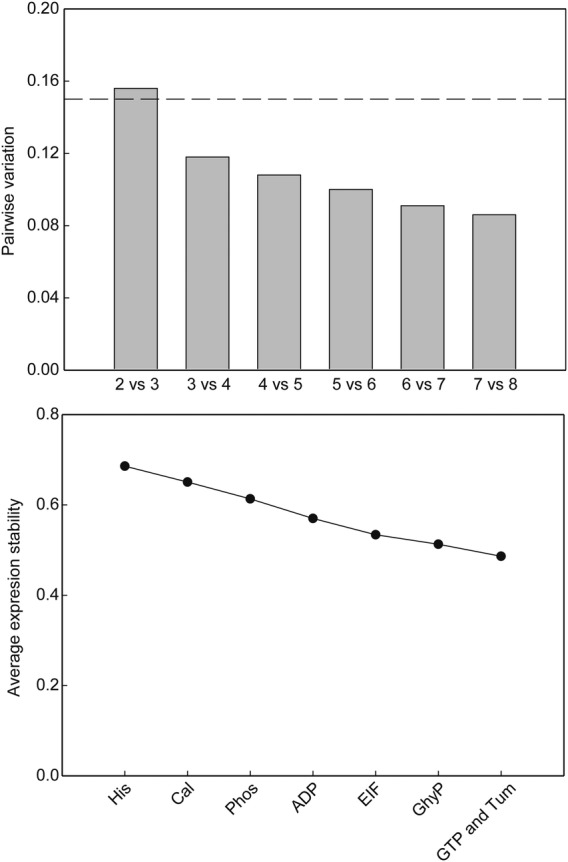
Results of pairwise variation analysis (upper panel) and average expression stability calculations (lower panel) undertaken using GeNorm from the quantitative PCR of alfalfa gene transcripts using primers designed for eight potential reference genes (Phos, phosphoprotein phosphatase type 2A; His, histone H3; Cal, calmodulin; ADP, ADP-ribosylation factor; EIF, elongation initiation factor; GhyP, glyceraldehyde-3-phosphate dehydrogenase; GTP, GTP-binding protein; and Tum, translationally controlled tumour protein). The broken horizontal line on the upper panel represents the 0.15 suggested cutoff for determining the optimum number of reference genes required for normalization.

The pairwise variation analysis undertaken with GeNorm identified that three reference genes would be required for the adequate normalization of transcript numbers for the genes of interest within this sample set (Fig. 8, upper panel). The candidate reference genes identified as being the most suitable for normalization under the current experimental conditions were GTP-binding protein, translationally controlled tumour protein and glyceraldehyde-3-phosphate dehydrogenase (Fig. 8, lower panel).

**Figure 9.**
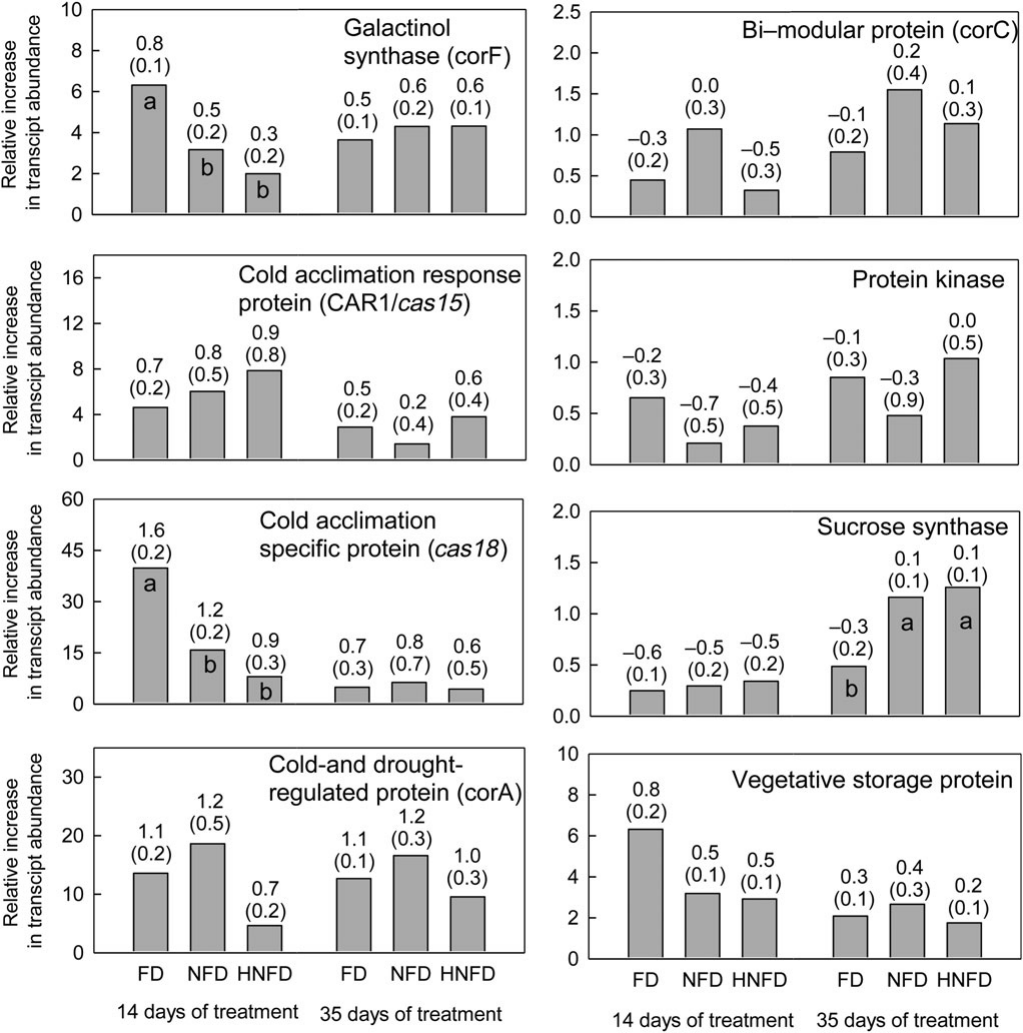
Relative increase in gene transcript abundance (relative to fully watered controls) of eight genes in the taproots of alfalfa cultivars (grouped into fall dormant (FD), non-fall dormant (NFD) or highly non-fall dormant (HNFD) groups based on their level of fall dormancy observed in the fall dormancy screening experiment) receiving 25 % of their water requirement. Values above the bars are the transformed means (log transformation) with the associated standard errors in parentheses. Bars with different letters within the same gene and number of days from treatment were identified as being different (*P* < 0.05) using ANOVA with non-orthogonal contrasts on transformed data.

Of the eight gene transcripts investigated, five (galactinol synthase: *corF*; cold acclimation responsive protein: CAR1/*cas15*; cold acclimation-specific protein: *cas18*; cold- and drought-regulated protein: corA; and the vegetative storage protein: VSP) increased in abundance relative to the fully watered controls when plants were exposed to a water deficit for both 14 and 35 days (Fig. 9). The increase in abundance of gene transcripts for galactinol synthase (*corF*) and cold acclimation-specific protein (*cas18*) differed between fall dormancy groupings (*P* < 0.05). There was a similar effect with VSP gene transcript abundance; however, this effect had a *P* value of 0.061. Both these gene transcripts had a greater abundance relative to the fully watered controls in the fall dormant groups compared with the non-fall dormant or highly non-fall dormant groups after 14 days of treatment. However, this effect was transient with no influence from fall dormancy grouping present at 35 days of treatment.

The abundance of *sucrose synthase* gene transcripts (relative to the fully watered controls) was lower in the fall dormant cultivars compared with the non-fall dormant and highly non-fall dormant cultivars after 35 days of treatment. The expression of the sucrose synthase gene at 14 days of treatment was not statistically different among the fall dormancy groupings.

## Discussion

The shoot growth of the water deficit stressed plants relative to fully watered controls at 14 days of treatment differed by fall dormancy grouping, with the fall dormant cultivars able to maintain shoot growth for longer periods under a water deficit (Fig. 5). Past research has noted that in the cool temperate environments of Australia that do not expose alfalfa crops to extreme winter temperatures, fall dormant cultivars outperform non-fall dormant cultivars in terms of forage yield in the face of mild to moderate drought (Pembleton *et al.* 2010*a*, *b*) despite having no need for the freezing tolerance that fall dormancy is associated with. Similarly, in certain temperate environments in China that receive nearly 1000 mm of annual precipitation, Wang *et al.* (2009) noted differences in annual forage yield among fall dormancy groupings during drought (600 mm annual rainfall) years but not during years receiving rainfall close to the long-term average. Fall dormancy group by environmental interactions was observed in North America as well, with superior performance of fall dormant cultivars in water-limited environments (Orloff and Hanson 2008). However, the confounding effects of both summer droughts and freezing winter temperatures make it difficult to attribute this expression of fall dormancy group by environmental interactions to the superior freezing or to the drought tolerance of fall dormant cultivars.

The fall dormant group also did not fully close their stomata until lower shoot water potentials were reached when compared with the highly non-fall dormant types. By the 35th day of treatment, there was no difference in relative shoot growth among the fall dormancy groupings and the consequences of a low shoot water potential on stomatal conductance were similar among the fall dormancy groups. The cumulative water deficit (water applied to the 100 % replacement water requirement treatment minus water applied to the 25 % replacement water requirement treatment) by this time was four times as great as the water deficit at 14 days (a 2530-mL deficit compared with a 625-mL deficit). This was reflected in plant shoot water potential for which there was no difference between the fully watered and the water deficit treated plants up to the 12th day of treatment while a 2.5-fold decrease from the fully watered plants occurred between 19 and 33 days of treatment. The timing of when the acclimation to water deficit was observed to occur in relation to the severity of the water deficit stress suggests that the specific acclimation to water deficit by more fall dormant cultivars of alfalfa is expressed early after the onset of water deficits and that more extended stress restricts growth equally for all cultivars. This observation is further supported by the difference between the fall dormancy groupings in the relationship between shoot water potential and stomatal conductance. The superior vigour of the fall dormant cultivars over non-fall dormant cultivars under a mild water deficit as indicated in Fig. 5 helped this fall dormancy grouping maintain stomatal conductance and leaf area expansion under lower shoot water potentials (Fig. 7). This would enable increased net plant photosynthesis and a further continuation of shoot growth in these cultivars in the face of mild to moderate water deficits, as has been previously observed under field conditions (Orloff and Hanson 2008; Wang *et al.* 2009; Pembleton *et al.* 2010*a*, *b*). A similar stomatal regulation strategy has been observed in the water deficit tolerant forage grass tall fescue (*Festuca arundinacea*) compared with the water deficit sensitive perennial ryegrass (*Lolium perenne*) (Holloway-Phillips and Brodribb 2011).

The maintenance of stomatal conductance, leaf area expansion and shoot growth at lower shoot water potentials would require osmotic adjustment within the plant. Osmotic adjustment is a key strategy that alfalfa plants use to minimize damage when exposed to freezing temperatures (Castonguay *et al.* 2006). The analysis of gene transcript abundance after 14 days of treatment identified that the transcripts for several genes that have been associated with the adaptation to freezing temperatures in alfalfa increased in abundance relative to the fully watered controls when exposed to a water deficit. The genetic basis of acclimation to freezing temperatures is common among all alfalfa fall dormancy groups (Castonguay *et al.* 1997). Consequently, the presence of freezing tolerance gene transcripts across all the fall dormancy groups as shown in Fig. 9 could be expected. After 14 days of water deficit, the *cas18* and *corF* gene transcripts had an increase in abundance that was related to fall dormancy grouping and their relative adaptation to water deficit indicated by the plant growth measurements. However, after 35 days of exposure to water deficit there was no pattern in gene transcript abundance that was related to fall dormancy and consequently water deficit tolerance. In Arabidopsis (*Arabidopsis thaliana*), wheat (*Triticum aestivum*), *Nicotiana benthamiana* and rice (*Oryza sativa*), homologues of the *cas18* and *corF* genes have been identified as early response genes following the exposure to a water deficit (Zhu 2002). The signalling pathways leading to the expression of these genes are constitutively maintained within the cell, leading to a rapid increase in their transcript abundance after exposure to stress (typical of dehydrins; Hanin *et al.* 2011). The other genes that exhibited an increase in transcript abundance are homologues of delayed response genes identified by Jaglo-Ottosen *et al.* (1998) and Kasuga *et al.* (1999). The signalling pathways for these genes need to be constructed before they are expressed (Zhu 2002). The differential expression of the early response genes observed at Day 14 but not at Day 35 of treatment suggests that although the non-fall dormant cultivars possess a functional form of the freezing tolerance genes, they may not have an as efficient signalling pathway for these genes in place compared with the fall dormant cultivars. This may partly explain the difference in adaptation at a plant growth/physiology level between fall dormancy groups treated to mild water deficit stress (14 days of treatment) but not to severe water deficit stress (35 days of treatment) as observed in this experiment. The creation of plants that under/overexpress genes may be required to fully understand the impact these genes and their signalling pathways have on the adaptation of plants to a water deficit.

The *cas18* gene transcript encodes for a protein that has considerable homology to dehydrins isolated from other species (Wolfraim *et al.* 1993; Close 1997). Dehydrins are thought to maintain cellular membrane integrity during dehydration (Close 1997; Hanin *et al.* 2011) and this has been the hypothesized role for the cas18 protein during freezing adaptation. Given the upregulation of this gene transcript in the drought-tolerant fall dormant cultivars when exposed to a water deficit, it is conceivable that the role of the cas18 protein during adaption to a water deficit is similar to that of dehydrins in other species.

The *corF* transcript encodes for galactinol synthase, a key enzyme in the formation of raffinose family oligosaccharides (RFOs) (Peterbauer and Richter 2001). The concentration of RFOs in alfalfa taproots has previously been correlated with freezing tolerance and winter survival (Castonguay *et al.* 1995; Cunningham *et al.* 2003), and an increase in the tissue concentration of RFOs has been associated with dehydration tolerance in *Xerophyta viscosa* (Peters *et al.* 2007) and Arabidopsis (Taji *et al.* 2002). Differences in the levels of *corF* gene transcripts in the taproots of alfalfa cultivars grouped into fall dormancy groups during the onset of fall dormancy have been noted by Cunningham *et al.* (2003). The role of RFOs in both freezing and water deficit stress tolerance may be through the protection of cellular structures or the stabilization of cellular membranes (Leopold 1990; Crowe *et al.* 1992).

The reduced abundance of the type 1 sucrose synthase gene transcripts in the water deficit tolerant fall dormant alfalfa cultivars after 35 days of treatment is novel given the requirement for increased concentrations of osmolytes in the cytosol when the plant is exposed to a water deficit. Furthermore, simple sugars, including sucrose, are thought to help stabilize membranes during freezing desiccation (Crowe and Crowe 1992; Sun *et al.* 1994). Interestingly, Sus1 in Arabidopsis was also reported to be induced by changes in leaf osmotic potential via an ABA-independent mechanism during low-temperature stress and osmotic stress (Déjardin *et al.* 1999). Whereas transcript levels of *Sus1* could increase several fold after osmotic stress exposures, researchers failed to observe any changes to the sucrose synthase protein levels on a western blot even after long-term cold and drought treatments. Déjardin *et al.* (1999) proposed that *Sus1* is under tight post-transcriptional control during osmotic stress and that *Sus1* mRNA might perhaps be produced and stored for use during specific stages of cold hardening or during plant recovery from the stress. In alfalfa the maintenance of taproot starch reserves has been linked with the long-term persistence in drought-prone environments (Boschma and Williams 2008). Given the role that sucrose synthase plays in utilizing the glucose released from starch degradation, the downregulation of this gene in the fall dormant cultivars could be a consequence of a long-term energy maintenance strategy under drought conditions in these cultivars. If true, this response indicates that fall dormant cultivars are not only more tolerant of mild water deficit conditions compared with more non-fall dormant cultivars but they may also be more persistent when exposed to severe long-term droughts. Further research is required to understand the role of sucrose synthase in alfalfa under different environmental conditions.

From the transcript abundance profiles and the punitive proteins these transcripts encode for, it can be concluded that the adaptation of the fall dormant cultivars is due to improved protection of cellular structures and membranes in the short term. The transcripts involved in stress signalling, protein storage and cellular regulation while also increased in abundance did not show any differential expression related to the adaptation of fall dormant cultivars to water deficit. The absence of differential expression of these transcripts is interesting as cross-talk between stress signalling pathways as well as the difference in cellular regulation has been linked to associations between abiotic stress tolerance including both freezing and drought (Zhu 2002; Seki *et al.* 2003; Chinnusamy *et al.* 2004). Consequently, if the drought tolerance of non-fall dormant alfalfa cultivars is to be improved without the use of gene modifications, focus should be placed on the improvement of the protective cellular structures and membranes by breeding together cultivars with desired gene expression patterns.

While this study is not the first to examine the responses of alfalfa cultivars to exposure to a water deficit (see Pembleton *et al.* 2009; Erice *et al.* 2011 for others), it is the first that has examined a broad enough range of fall dormancy to be able to conclude that decreasing levels of fall dormancy reduce adaptation to mild water deficit conditions. In support of this finding, transcripts of two genes that are associated with freezing tolerance increased in abundance in the more fall dormant types when exposed to a mild water deficit, while the transcript of a type 1 sucrose synthase gene associated with phloem loading and carbon channelling in roots decreased when the fall dormant types were exposed to a severe water deficit. The identification of a partial separation between fall dormancy and freezing tolerance by Brouwer *et al.* (2000) and Brummer *et al.* (2000) along with the linkage between freezing tolerance and water deficit tolerance at a molecular level identified in this study suggests that it will be possible to achieve greater drought tolerance in non-fall dormant cultivars. This study has identified that alfalfa cultivars must be of the fall dormant type to exhibit superior adaptation to a water deficit. The stress adaptation pathways associated with the upregulation of the freezing tolerance genes *cas18* and *corF* during mild water deficit stress and the downregulation of a type 1 sucrose synthase gene during severe water deficit stress appear to be the molecular mechanism by which fall dormant cultivars of alfalfa ‘give drought the cold shoulder’.

## Sources of Funding

This work was supported by Dairy Australia Ltd and the Minister for Agriculture, Fisheries and Forestry Awards of the 2011 Department of Agriculture, Fisheries and Forestry (DAFF) Science and Innovation Awards for Young People in Agriculture, Fisheries and Forestry.

## Contributions by the Authors

K.G.P. conceived the study, designed the experiments and carried out the experiments. P.S. helped design and supervised the qRT-PCR analysis. Both authors drafted the paper and approved the final draft.

## Conflict of Interest Statement

None declared.
